# Low Temperature Fast‐Charging Li Ion Batteries Enabled by f‐Orbital Hybridization Induced TiNb_2_O_7_ Electronic Structure

**DOI:** 10.1002/advs.202504808

**Published:** 2025-06-20

**Authors:** Anran Shi, Lichao Tan, Xiumei Song, Shenglu Geng, Wenting Li, Jiayi Wang, Kai Zong, Lin Yang, Dan Luo, Shuaifeng Lou, Xin Wang, Biqiong Wang, Zhongwei Chen

**Affiliations:** ^1^ Institute of Carbon Neutrality Zhejiang Wanli University Ningbo 315100 China; ^2^ State Key Laboratory of Space Power‐Sources School of Chemistry and Chemical Engineering Harbin Institute of Technology Harbin 150001 China; ^3^ China Tower Corporation Limited No.9 Dongran North Street, Haidian District Beijing 100089 China; ^4^ Power Battery and System Research Center State Key Laboratory of Catalysis Dalian Institute of Chemical Physics Chinese Academy of Sciences Dalian 116023 China; ^5^ School of Chemistry and Chemical Engineering Yangzhou University Yangzhou 225009 China

**Keywords:** fast charging, lanthanide regulating, Li‐ion batteries, low temperture, TiNb_2_O_7_

## Abstract

Li‐ion batteries for electric vehicles and aviation require fast charging, long cycle life, and a wide operating temperature range. However, the lack of anode materials that offer both high capacity and stability at high charging/discharging rates significantly impedes their development. Herein, the introduction of lanthanide with f‐Orbital electronic configurations widens the ion transport channels and significantly alters the original electronic structure, leading to a notable improvement in acceleration kinetics. X‐ray absorption spectroscopy is employed to depict lanthanide earth elements that can lower lattice strain and accelerate the diffusion of Li^+^. The Tm_0.01_‐TNO delivers an outstanding specific capacity of 150.9 mAh g^−1^ at 50 C. Even at low temperatures (−30 °C), Tm_0.01_‐TNO exhibits stable cycling performance with 100% capacity retention over 500 cycles at 1 C. This work demonstrates an enormous promise for scalability in practical low‐temperature applications.

## Graphical abstract

This work provides an excellent diagram for lanthanide‐regulating engineering toward a notable improvement in acceleration kinetics, employing to depict rare earth elements that can lower lattice strain and accelerate the diffusion of Li^+^, holding great potential for the development of practically viable Li ion batteries, and also enlightening the material designs in the related low‐temperature anode.

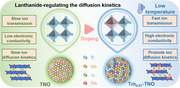

## 1 Introduction

As the energy storage battery market demands increasingly higher performance in terms of range and low‐temperature operation for lithium‐ion batteries, the traditional graphite anode materials struggle with slow kinetics under high power and low‐temperature conditions.^[^ ^1^, ^2^, ^3^ ^]^ Additionally, the low potential (≈0.2 V) of the embedded lithium makes these materials susceptible to the formation of lithium dendrites during rapid charging and discharging, which can lead to irreversible lithium loss and raise safety concerns for the batteries.^[^ ^4^, ^5^, ^6^, ^7^ ^]^

Due to its special crystal structure, the TiNb_2_O_7_ (TNO) experiences only a 3.6% change in cell volume during the embedding and de‐embedding process of Li^+^, which contributes to its excellent stability and highly reversible cycling performance.^[^ ^8^, ^9^, ^10^ ^]^ Furthermore, the embedding and de‐embedding process of Li^+^ involves the transfer of 5 electrons, corresponding to the redox reaction with 5 electron pairs,^[^ ^11^, ^12^ ^]^ which gives TiNb_2_O_7_ a theoretical specific capacity of up to 387.6 mAh g^−1^. In addition to the above, the high discharge potential of the TNO material (≈1.6 V) effectively helps prevent the formation of solid electrolyte interphase (SEI) and lithium dendrites, significantly enhancing safety performance, even at low temperatures.^[^ ^13^, ^14^ ^]^ The poor electronic conductivity of the TNO negative electrode, which is a consequence of its intrinsic safety properties, restricts its wide application at low temperatures.^[^ ^15^ ^]^

Unlike conventional d or p‐block elements, lanthanide elements (LE) with 4f^x^5d^y^6s^2^ ground states and local 4f orbitals can stimulate the formation of defect states within the host material's band gap and localize the expansion of the crystal structure, resulting in optimized ion transport dynamics.^[^ ^16^, ^17^, ^18^ ^]^ Furthermore, the localized 4f orbital can hybridize with the d/p/s orbital, which affects the electrical conductivity by narrowing the band gap and creating an impurity band of LE elements that span across the Fermi level.^[^ ^19^, ^20^, ^21^ ^]^

Herein, we report an in situ doping engineering method to tune the bandgap, thereby enhancing ion diffusion kinetics at both room and low temperatures. The expansion of crystal lattices, induced by the predistortion of the Nb─O bond, enhances the intercalation speed and reduces volume expansion during Li^+^ intercalation. DFT calculations further demonstrate that in situ doping changes the electronic environment of the material, promoting electron transport and ion diffusion. This results in high lithium diffusion coefficients and improved fast‐charging capability low‐temperature. As a consequence, the Tm_0.01_‐TNO exhibited excellent rate capability with the preserving of a remarkable capacity of 150.9 mAh g^−1^ under 50 C. Even at −30 °C, the Tm_0.01_‐TNO delivered a significant reversible capacity (161.2 mA h g^−1^) and inspiring durability (100% after ≈500 cycles at 1 C).

## 2 Results and Discussion

**Figure 1:**
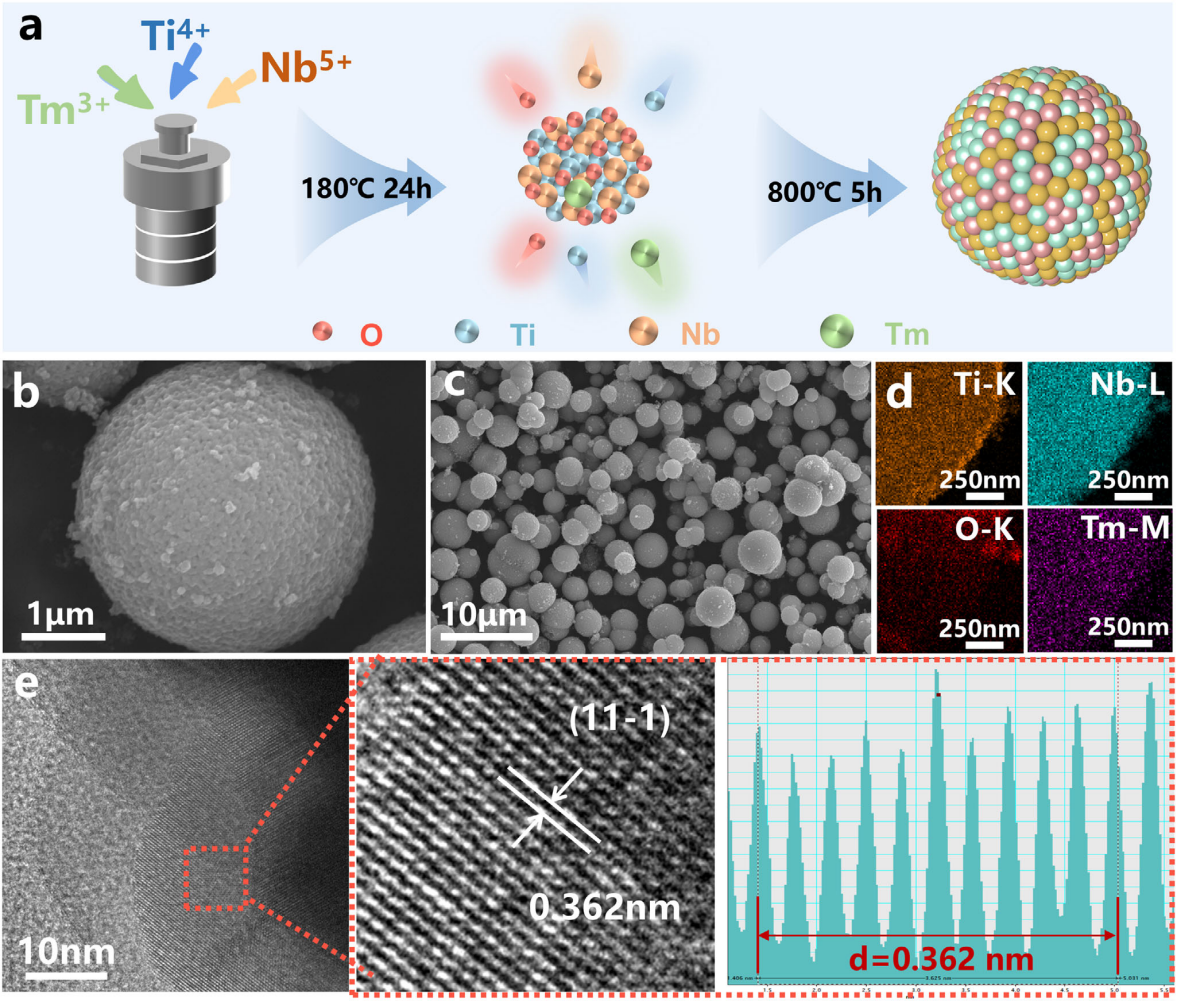
a) Schematic diagram of the synthesis process of Tm_0.01_‐TNO, b,c) SEM images, d) EDS mappings of the Tm_0.01_‐TNO, e) HR‐TEM image, and STEM‐HADDF image of Tm_0.01_‐TNO.

Typically, the TNO precursor was fabricated by a facile hydrothermal strategy, and the heterostructured Tm_0.01_‐TNO microsphere is obtained after in situ high‐temperature polymerization, as shown in **Figure** **1**a. The scanning electron microscopy (SEM) images at different ratios (Figure 1b,c) reveal that nanoparticles accumulate and form a spherical structure. The EDS elemental mapping (Figure 1d) of Tm_0.01_‐TNO confirms the uniform distribution of Ti, Nb, O, and Tm elements, implying the successful incorporation of Tm. As shown in the high‐resolution transmission electron microscopy (HR‐TEM) image (Figure 1e), the measured lattice fringes are in the size of 0.362 nm, which can be ascribed to the TNO (11‐1) plane.^[^ ^22^ ^]^

**Figure 2:**
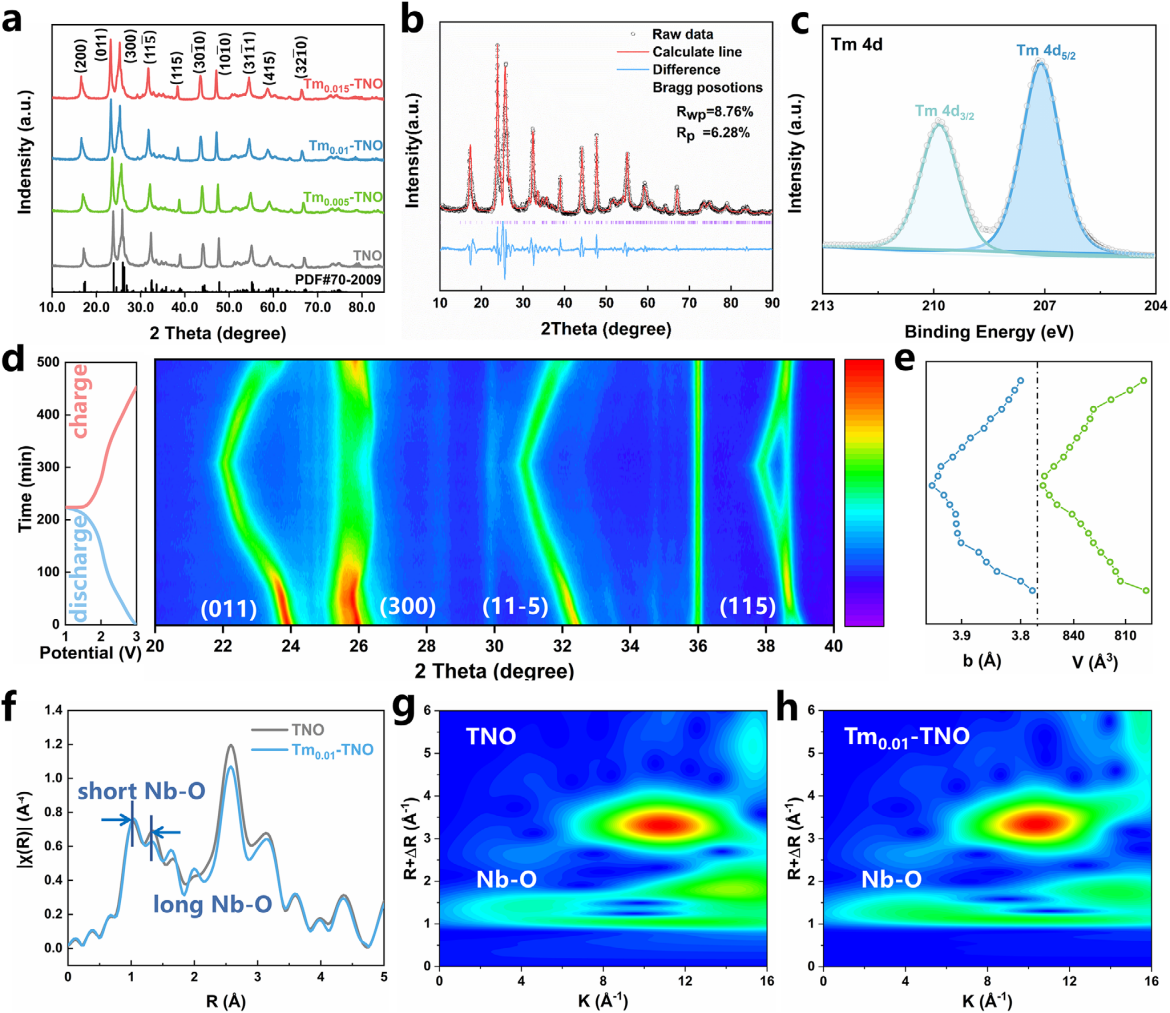
a) XRD patterns of TNO precursor, Tm_0.005_‐TNO, Tm_0.01_‐TNO and Tm_0.015_‐TNO, b) Refined XRD pattern of Tm_0.01_‐TNO, c) High‐resolution XPS spectra of Tm 4d in Tm_0.01_‐TNO, d) In situ XRD patterns of Tm_0.01_‐TNO with corresponding discharge/charge curves and e) lattice‐parameter variations, f) Extended X‐ray absorption fine structure (EXAFS) spectra of the Tm_0.01_‐TNO and TNO, g,h) WT‐transform contour plots at the Nb k‐edge of Tm_0.01_‐TNO and TNO.

X‐ray diffraction (XRD) patterns (**Figure** **2**a) of TNO, Tm_0.005_‐TNO, Tm_0.01_‐TNO, and Tm_0.015_‐TNO anode materials all show the peaks of the TNO materials^[^ ^23^ ^]^ (PDF#70‐2009). Meanwhile, the observed two high broad peaks at 23.1° and 27.2° are attributed to the (011) and (300) planes of TNO, respectively.^[^ ^24^, ^25^ ^]^ The Rietveld refinements of XRD data (Figure 2b; Figure S1, Supporting Information) reveal that the unit cell volume of Tm_0.01_‐TNO (798.100 Å^3^) is larger than TNO (795.593 Å^3^). This increase is attributed to the expansion of interplanar spacing induced by Tm doping. The structure of the Tm_0.01_‐TNO was further characterized by X‐ray photoelectron spectroscopy (XPS). The survey spectrum in Figure S2 (Supporting Information) shows that the Tm_0.01_‐TNO is composed of Ti, Nb, O, and Tm elements. The Tm 4d spectrum of the Tm_0.01_‐TNO exhibits two peaks at 207.3 and 209.2 eV, which are attributed to Tm^3+^, indicating the valence state of Tm^[^ ^18^ ^]^ (Figure 2c). This observation is further supported by a slight negative shift of the Nb (Figure S3a, Supporting Information) and Ti (Figure S3b, Supporting Information) spectra, suggesting that the introduction of the Tm element induces charge redistribution and internal electron transfer within the material. To examine the crystal structure evolution and stability of the Tm_0.01_‐TNO, in situ XRD was performed during the initial Li^+^ insertion/extraction between 1–3 V. The diffraction peak of Tm_0.01_‐TNO, which is centered at 23.8°, depicts a zigzag evolution during the lithiation process. Meanwhile, the peaks corresponding to the (011), (11‐5), and (115) planes gradually shift to small angles and decrease in intensity, indicating a reduction in the orderliness of the crystal structure (Figure 2d). The Rietveld refinements were conducted on the in situ XRD data to understand the changes in the lattice constant better. The observations confirm that the introduction of large ionic radius elements mitigated volume expansion during Li^+^ intercalation. Upon Li^+^ removal, the crystal lattice contracted, causing the diffraction peaks to alter backward, indicating high electrochemical reversibility.

To further investigate the elemental chemical states and electronic configuration of Tm_0.01_‐TNO, X‐ray absorption spectroscopy was utilized. The electronic structure changes after Tm integration were identified using an X‐ray absorption near‐edge structure (XANES) spectrum. Figure S4 (Supporting Information) shows that the Nb K‐edge spectra of Tm_0.01_‐TNO are similar in shape to those of standard TNO,^[^ ^26^ ^]^ indicating that Nb exists in a similar state in both Tm_0.01_‐TNO and TNO. This result suggests that Tm plays a role in electron donation in the unit, leading to modification in the electronic state of Nb. The extended X‐ray absorption fine structure (EXAFS) results (Figure 2e) show similar R space distribution of Tm_0.01_‐TNO and TNO, demonstrating the concurrent existence of both short and long Nb─O scattering in conventional Tm_0.01_‐TNO and TNO.^[^ ^27^ ^]^ Significantly, the ratio of long Nb─O scattering in Tm_0.01_‐TNO is lower than that in conventional TNO, which is due to the more uniform dispersion of Nb ions in Tm_0.01_‐TNO. The EXAFS wavelet transformation (WTs) analysis (Figure 2f,g) confirms that the coordinating environment around Nb in Tm_0.01_‐TNO is comparable to that in TNO. The intensity maximum values at 6.8 and 5.3 Å^−1^ are ascribed to the Nb─O bond in the WTs contour plots of TNO and Tm_0.01_‐TNO samples,^[^ ^28^ ^]^ respectively. These results validate the adjustment of asymmetric coordination in the Nb─O bonds due to Tm modification.

**Figure 3:**
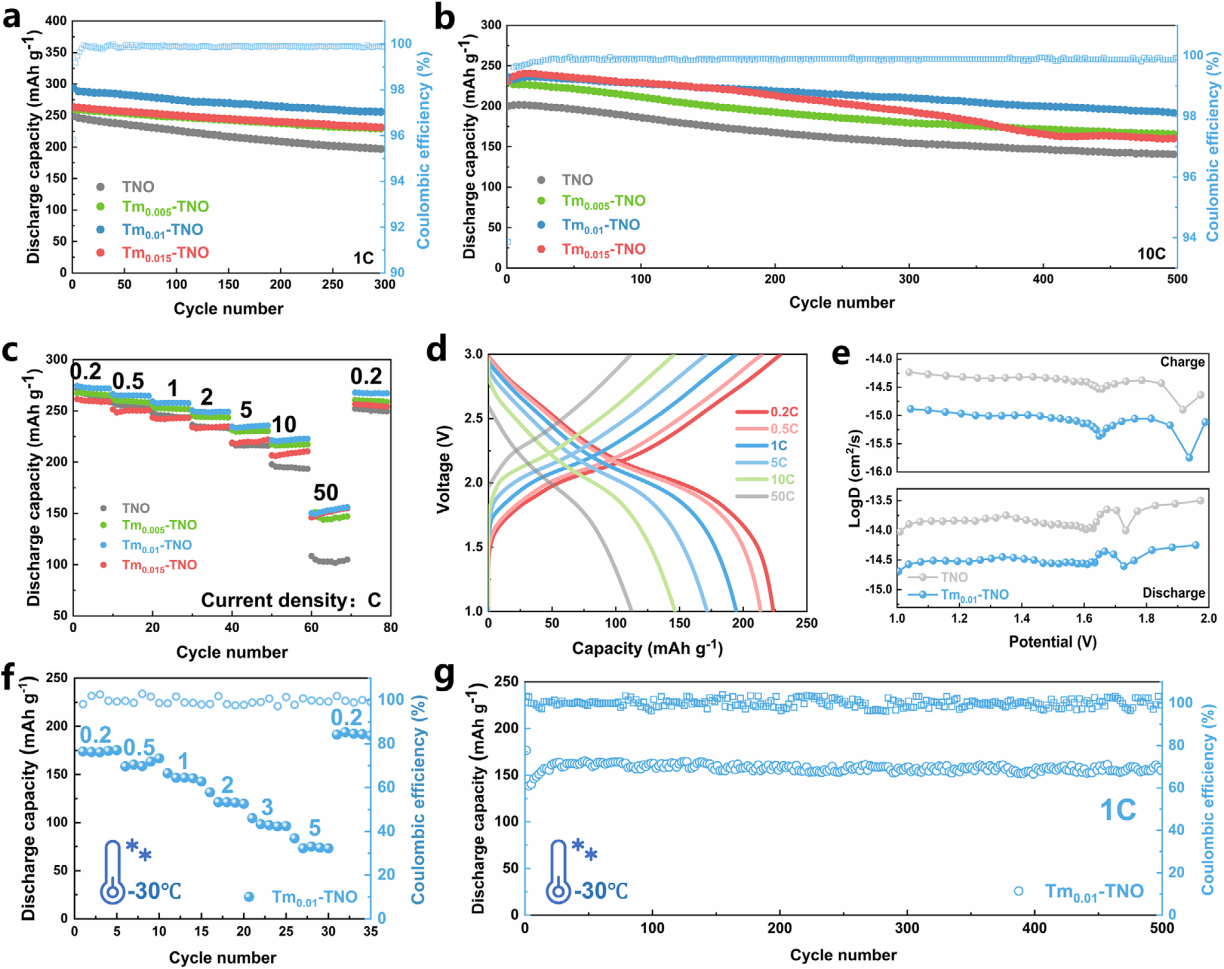
a,b) Long‐term cycling stability of Tm_0.01_‐TNO and TNO at 1 C and 10 C, c) Rate capability of Tm_0.01_‐TNO and TNO, d) Galvanostatic charge/discharge profiles of Tm_0.01_‐TNO, e) Li^+^ diffusion coefficients of the Tm_0.01_‐TNO and TNO, f) Rate performances of Tm_0.01_‐TNO operated at ‐30 °C, g) Long‐term cycling stability of Tm_0.01_‐TNO at ‐30 °C.

Besides above, excellent cycling reliability of the Tm_0.01_‐TNO electrode was demonstrated at 1 C (**Figure** **3**a), with a high reversible capability of 289.5 mAh g^−1^ and capacity retention of 84.4% over 500 cycles, outperforming the TNO (248.2 mAh g^−1^, 72.9%), Tm_0.005_‐TNO (260.3 mAh g^−1^, 83.5%), and Tm_0.005_‐TNO (263.9 mAh g^−1^, 82.4%). The Tm_0.01_‐TNO electrode maintained a high capacity of 235.7 mAh g^−1^ with a capacity loss of just 0.0655% per cycle over 1000 cycles when the current rate was raised to 10 C (Figure 3b). The high cycle stability and capacity enhancement of Tm_0.01_‐TNO can be attributed to the ion‐filling effect of Tm‐doping, which enlarges the interlayer distance for lithium diffusion. In addition, the Tm_0.01_‐TNO electrode capacity to fast‐charging across a current density range of 0.2–50 C was investigated (Figure 3c,d). Notably, the Tm_0.01_‐TNO electrode is still able to reach a steady reversible capacity of 150.9 mAh g^−1^ at a high current density of 50 C, surpassing that of TNO, Tm_0.005_‐TNO, and Tm_0.015_‐TNO. The high kinetic reversibility is further demonstrated by the electrode's ability to restore its reversible capacity to the initial level even when the rate is reduced to 0.2 C. This performance is attributed to the improved ion diffusion and electron transfer facilitated by the electron compensation effect of Tm doping. To gain a comprehensive understanding of the interface electrochemistry of Tm_0.01_‐TNO, sweep‐rate‐dependent CV measurements were performed to reveal the behavior of lithium storage. As shown in Figure S5a,b (Supporting Information), the kinetic behaviors of TNO and Tm_0.01_‐TNO, rate‐dependent CV measurements were performed at scan rates from 0.1 to 1.0 mV s^−1^. As the sweep rate increases, the primary oxidation peaks shift to higher potentials, while the reduction peaks shift to lower potentials. At a sweep rate of 0.1 mV s^−1^, the capacitive contribution accounts for 75.2% of the total capacity. As the scan rate increases, the capacitive contribution rises over time, reaching a maximum value of 89.9% at 1.0 mV s^−1^ (Figure S6a, Supporting Information). Under the same conditions, the capacitive ratio of TNO also increased from 60.2% to 77.1%, but it remained lower than that of the Tm_0.01_‐TNO (Figure S6b, Supporting Information). This high capacitance contribution is attributed to the fast lithium storage at high operating currents, which is achieved through the electron compensation effect induced by Tm. The macroscopic diffusion coefficients were measured using galvanostatic intermittent titration techniques to assess the intrinsic ion‐transport capabilities of Tm_0.01_‐TNO. Figure 3e shows that in both charging and discharging modes, Li diffusion in Tm_0.01_‐TNO is substantially faster than in TNO. Tm_0.01_‐TNO was evaluated for its cycling and multiplication at low temperatures (−30 °C) to assess its enhanced rate capabilities. The low‐temperature rate performance of Tm_0.01_‐TNO (Figure 3f) demonstrates its high capacity at current densities ranging from 0.2 C to 5 C. At −30 °C, Tm_0.01_‐TNO maintains a stable capacity of 161.2 mAh g^−1^ and achieves a high‐capacity retention of 100% after 500 cycles at 1 C, demonstrating exceptional low‐temperature capacity retention.

**Figure 4:**
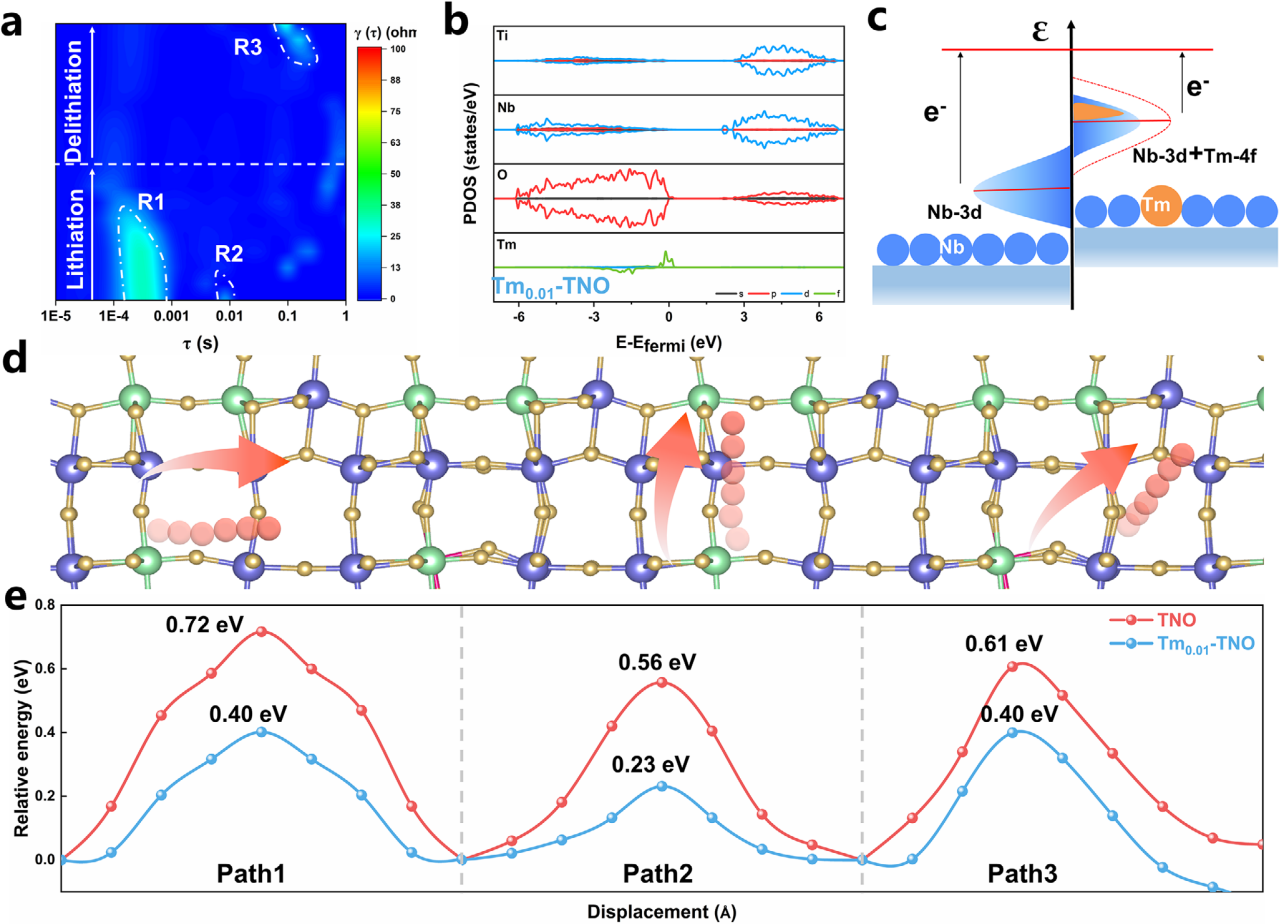
a) In situ DRT pattern of Tm_0.01_‐TNO, b) Calculated PDOS values of Tm_0.01_‐TNO, c) Proposed mechanism for Tm‐induced electronic structure adjustment of Nb‐based materials d) Schematic illustration of lithium‐ion diffusion routes in TNO crystal structures, e) Comparison of Li^+^ diffusion energy barriers between the Tm_0.01_‐TNO and TNO under the three paths.

EIS is subsequently investigated using a distribution of relaxation times (DRT) approach in order to determine the chemical stages involved in the lithiation process.^[^ ^29^, ^30^ ^]^ Peaks R1 and R2, with shorter relaxation durations, are ascribed to interfacial processes, whereas peak R3 indicates the charge‐transfer reactions at the anode. R1, R2, and R3 are the initials of the three prominent peaks that emerged in the in situ DRT findings after discharging (Figure S7, Supporting Information). Before lithiation, Tm_0.01_‐TNO exhibits a large R1 peak due to the poor conductivity of the material, while after lithiation, the R1 peak becomes weaker. After delithiation, the weak R1 peaks are still maintained, with the existence of fewer R2 peaks compared to the TNO (**Figure** **4**a; Figure S8, Supporting Information). These results are due to the formation of an SEI film during the charging and discharging processes. As shown by the calculated total/partial density of states (Figure 4b; Figure S9, Supporting Information), the simulated band gap of TNO is ≈2.35 eV, higher than Tm_0.01_‐TNO (1.66 eV). After the introduction of Tm atoms, the band gap narrows, and an impurity band of Tm forms near the Fermi level, indicating that Tm0.01‐TNO has superior conductivity due to the hybridized electronic states of Tm. As shown in Figure 4c, Tm─O has more ionic properties than Nb─O, which is due to more localized 4f electronic properties in Tm. Additionally, electronic states alter up with the coupling of 3d‐4f orbits, and 4f states can minimize electron modulation of electron‐hole recombination in bandgap semiconductors by accelerating electron transport due to their shared higher energy level. Therefore, the electrochemical performance of Tm_0.01_‐TNO would be positively impacted by the narrowing of the band gap of TNO. Subsequently, simulations of three lithium‐ion diffusion paths in the TNO material were conducted to verify that the introduction of the Tm element positively affects the crystal structure (Figure 4d). By comparing the diffusion energy barriers of lithium ions across three different migration paths, it is observed that the introduction of a large ionic radius effectively reduces the diffusion energy barrier and enhances the diffusion rate (Figure 4e).

**Figure 5:**
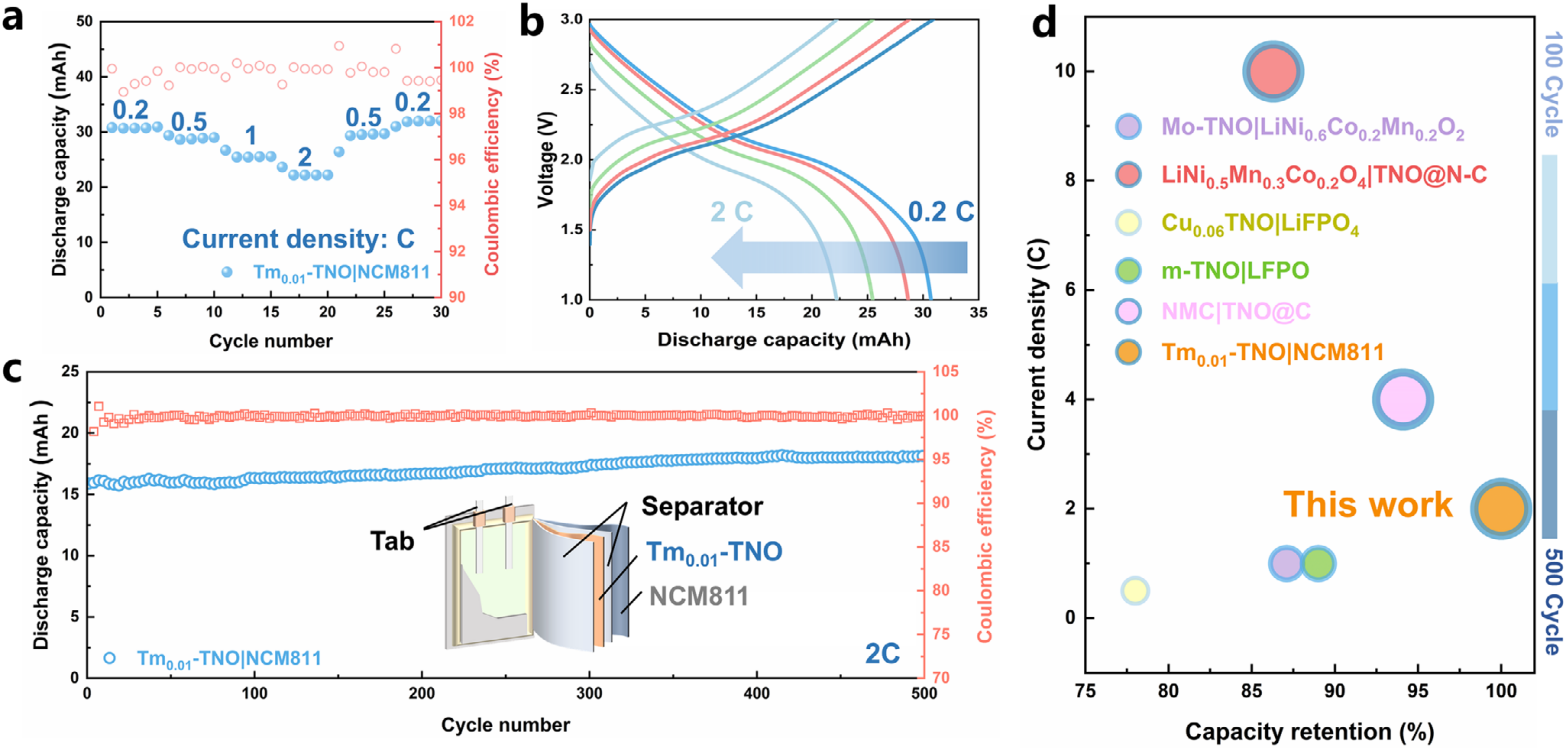
a) Rate capability of Tm_0.01_‐TNO |LiNi_0.8_Co_0.1_Mn_0.1_O_2_ pouch cell, b) Galvanostatic charge/discharge profiles of Tm_0.01_‐TNO |LiNi_0.8_Co_0.1_Mn_0.1_O_2_ pouch cell, c) Long‐term cyclability of Tm_0.01_‐TNO |LiNi_0.8_Co_0.1_Mn_0.1_O_2_ pouch cell, d) Comparison of the capacity retention and the cycle number for pouch cell in this work with TNO reported in literature.^[^ ^22^, ^31^, ^32^, ^33^, ^34^ ^]^

To confirm its potential commercial application, a pouch cell with Tm_0.01_‐TNO |LiNi_0.8_Co_0.1_Mn_0.1_O_2_ (**Figure** **5**a) was subsequently assembled. The cell demonstrates discharge capacities of 31.8, 28.7, 25.5, and 22.2 mAh at current rates of 0.2 C, 0.5 C, 1 C, and 2 C, respectively (Figure 5b). A reversible capacity of 31.9 mAh with a capacity retention of 100% can be achieved when the current density is reduced to 0.5 C. Figure 5c shows that the pouch cell retains a capacity of 18.2 mAh at 2 C after 500 cycles, surpassing that of a majority of reported TNO‐based electrodes^[^ ^22^, ^31^, ^32^, ^33^, ^34^ ^]^ (Figure 5d). These results reveal that Tm_0.01_‐TNO, with its expanded lattice spacing, offers promising performance as an anode material for high‐rate lithium‐ion batteries.

## 3 Conclusion

In summary, we succeeded in formulating a Tm_0.01_‐TNO anode material with a tailored electrical environment by utilizing in situ doping. XANES analysis reveals that the in situ doping of the Tm_0.01_‐TNO crystal structure results in the deformation of Nb─O bonds, which facilitates fast lithium‐ion transport at low temperatures. Based on DFT calculations, the addition of the Tm element significantly improves reaction kinetics and electronic conductivity by spontaneously tuning the electronic environment and inducing impurity energy bands at the Fermi energy level. Therefore, Tm_0.01_‐TNO exhibits a high reversible capacity, good rate performance, and low‐temperature capacity retention. The Tm_0.01_‐TNO electrode delivered a high‐rate capacity of 175.9 mAh g^−1^ at 50 C. Even at −30 °C, this electrode achieved a super high capacity of 174.5 mAh g^−1^ at 0.2 C and ultra‐long cycling stability with 100% retention after 500 cycles. This work provides new insight into the design of TNO‐based anode materials for high‐rate performance, low‐temperature batteries.

## Conflict of Interest

The authors declare no conflict of interest.

## Supporting information



Supporting Information

## Data Availability

The data that support the findings of this study are available from the corresponding author upon reasonable request.
