# Supplementary material for: Low Temperature Fast‐Charging Li Ion Batteries Enabled by f‐Orbital Hybridization Induced TiNb2O7 Electronic Structure
Source: Adv Sci (Weinh). 2025 Jun 20;12(34):e04808. doi: 10.1002/advs.202504808 (PMC12442630; doi:10.1002/advs.202504808)
Supplement: Supplementary file 1 — Supporting Information [file ADVS-12-e04808-s001.docx]

Supporting Information

**Low Temperature Fast-charging Li ion Batteries Enabled by f-Orbital Hybridization Induced TiNb_2_O_7_ Electronic Structure**

Anran Shi ^1, 2^, Lichao Tan ^1, *^, Xiumei Song ^1^, Shenglu Geng ^2^, Wenting Li ^5^, Jiayi Wang ^1^, Kai Zong ^1^, Dan Luo ^4^, Shuaifeng Lou ^2^, Xin Wang ^1, *^, Biqiong Wang ^3, *^, Zhongwei Chen ^4, *^

((Optional Dedication))

A. Shi, Prof. L. Tan, X. Song, J. Wang, K. Zong, Prof. X. Wang

^1^ Institute of Carbon Neutrality, Zhejiang Wanli University, Ningbo 315100, China

E-mail: tanlc@zwu.edu.cn; [wangx@zwu.edu.cn](mailto:wangx@zwu.edu.cn)

A. Shi, S. Geng, Prof. S. Lou

^2^ State Key Laboratory of Space Power-Sources, School of Chemistry and Chemical Engineering, Harbin Institute of Technology, Harbin 150001, China

Prof. B. Wang

^3^ China Tower Corporation Limited, No.9 Dongran North Street, Haidian District, Beijing 100089, China

E-mail: [wangbq9@chinatowercom.cn](mailto:wangbq9@chinatowercom.cn)

Prof. D. Luo, Prof. Z. Chen

^4^ Power Battery and System Research Center, State Key Laboratory of Catalysis, Dalian Institute of Chemical Physics, Chinese Academy of Sciences, Dalian 116023, China

E-mail: zwchen@dicp.ac.cn

^5^ School of chemistry and chemical Engineering, Yangzhou University, Yangzhou, 225009, China

E-mail: wtlichem@yzu.edu.cn

**Keywords:** Fast Charging, Low Temperture, TiNb_2_O_7_, Lanthanide Regulating, Li-ion Batteries

**Contents**

[**Experimental** S3](#_Toc64507480)

[**Figure S1**. Refined XRD pattern of TNO. S6](#_Toc64507480)

[**Figure S2**. The full XPS spectra of Tm_0.01_-TNO. S7](#_Toc64507480)

[**Figure S3**. XPS spectra of TNO and Tm_0.01_-TNO for Ti 2p (a); XPS spectra of TNO and Tm_0.01_-TNO for Nb 3d (b). S8](#_Toc64507481)

[**Figure S4**. X-ray absorption near edge spectroscopy (XANES) spectra of the Nb K-edge of the TNO and Tm_0.01_-TNO. S9](#_Toc64507482)

[**Figure S5**. CV curves of TNO (a) and Tm_0.01_-TNO (b) electrode at various scan rates. S10](#_Toc64507483)

[**Figure S6.** The percentage of pseudocapacitive contribution at different scan rates of of TNO (a) and Tm_0.01_-TNO (b). S11](#_Toc64507484)

[**Figure S7**. In-situ DRT pattern of TNO. S12](#_Toc64507485)

[**Figure S8**. Calculated PDOS values of TNO. S13](#_Toc64507486)

**Experimental**

**Materials preparation** The Tm_x_-TNO (x=0, 0.005, 0.01, 0.015, x represents the number of moles of Tm atoms) were prepared by a facile solvothermal approach. Tetrabutyl titanate (TBT, ≥99.0%) and Thulium chloride (TmCl_3_, 99.9%) were used as starting materials with a molar ratio of 1:0.005, 1:0.01 and 1:0.015, respectively. Typically, 50 mL of ethanol was ultrasonically vibrated for 1 hour while a suitable amount of TmCl_3_ was dissolved in it. Thereafter, 2.5 mmol TBT and 5 mmol niobium chloride were added to the above solution and stirred for 5 hours. The mixed solution was transferred to a Teflon-linked steel autoclave and preserved at 180°C for 24 h. After centrifuging the resultant white precipitate, it was dried under vacuum at 80 °C for 10 h. The precursor was then heated at 800 °C with a heating rate of 5 °C·min^-1^ for 5 h in air and natural cooling and finally obtained. TNO, Tm_0.005_-TNO, Tm_0.01_-TNO, Tm_0.015_-TNO.

**Materials characterizations** The crystalline structures of the composites were performed by X-ray diffraction (XRD, Bruker D8 Advance) with Cu Kα radiation. X-ray photoelectron spectroscopy (XPS) datum of the powers was examined via PHI 5700-ECSA System. Raman spectra were performed by applying a Labram high-resolution spectrometer (a wavelength of 532 nm). To observe the morphologies and structures of the materials, a scanning electron microscope (SEM, Helios Nanolab 600i) and transmission electron microscope (TEM, TecnaiG2F20) were determined in this paper.

**Electrochemical measurements** Preparation of half-cell: The working electrodes were comprised of the active composites, Super P, and polyvinylidene fluoride binder (8:1:1 in weight) mixed in N-methyl-2-pyrrolidone, followed by the slurry pasted on copper foils and dried at 80 °C for 10 h in a vacuum. The loading mass of the single-electrode was in the range of 1.2-1.4 mg·cm^-2^. The coin cells (CR2032) were assembled using Li foil as the counter, and LiPF_6_ (1 M) in a mixture of EC/ DEC/ DMC (1:1:1in volume) as the electrolyte. The Neware-CT3008 system was utilized to operate the galvanostatic charge/discharge tests in the range of 1.0-3.0 V and Galvanostatic intermittent titration technique (GITT). Electrochemical impedance spectroscopy (EIS) and cyclic voltammetry (CV) were performed on a CHI760 electrochemical workstation.

Preparation of pouch-cell: The pouch cells were operated by using Tm_0.01_-TNO as the negative, and commercial LiNi_0.8_Co_0.1_Mn_0.1_O_2_ as the positive. For the LiNi_0.8_Co_0.1_Mn_0.1_O_2_|Tm_0.01_-TNO full cell, the loading mass for the anode and the cathode electrode are 8 and 9 mg·cm^-2^ and 13 and 14 mg·cm^-2^, respectively. The optimized negative/positive areal capacity ratio of cathode/anode was 1/1.03.

**Computational methods** Density functional theory (DFT) calculations were performed by using the Vienna Ab-Initio Simulation Package (VASP) code[1]. The projector augmented wave (PAW)[2] method was used to describe the ionic cores. And the electron exchange-correlation was modeled by Perdew-Burke-Ernzerhof (PBE) function within generalized gradient approximation (GGA)[3]. A cut-off energy of 450 eV was used for the plane-wave basis set. The convergence criterion was 10^-4^ eV for energy and 0.05 eV/Å for force. And the Monkhorst-Pack k-point meshes were set as 2×2×2 for all calculations. The 1×3×2 TNO supercell was created, and 1 Ti atom in this supercell was substituted by Tm atom respectively to simulate Tm doped TNO.





**Figure S1.** Refined XRD pattern of TNO.


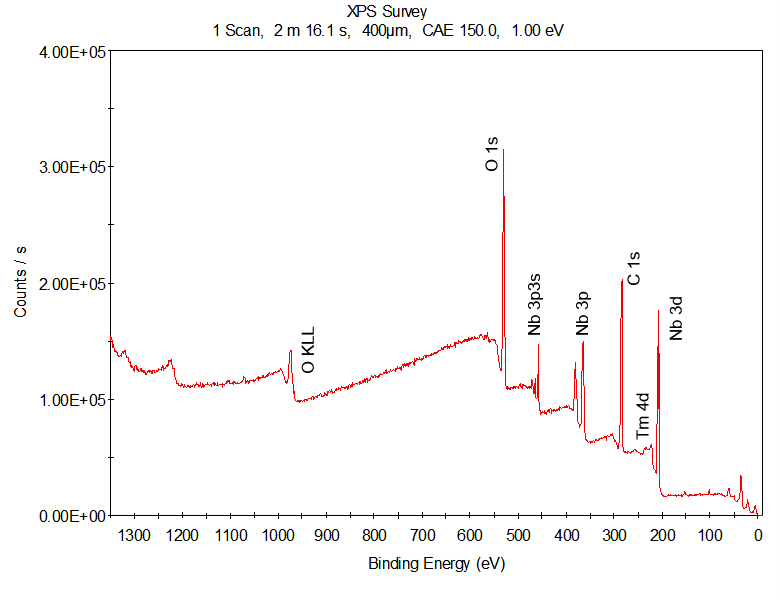


**Figure S2**. The full XPS spectra of Tm_0.01_-TNO.


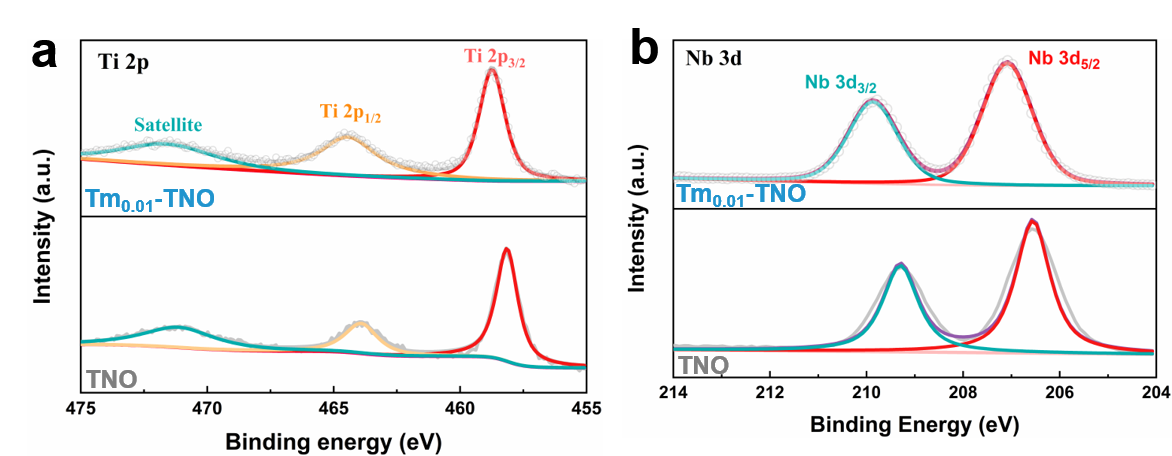


**Figure S3**. XPS spectra of TNO and Tm_0.01_-TNO for Ti 2p (a); XPS spectra of TNO and Tm_0.01_-TNO for Nb 3d (b).





**Figure S4**. X-ray absorption near edge spectroscopy (XANES) spectra of the Nb K-edge of the TNO and Tm_0.01_-TNO.


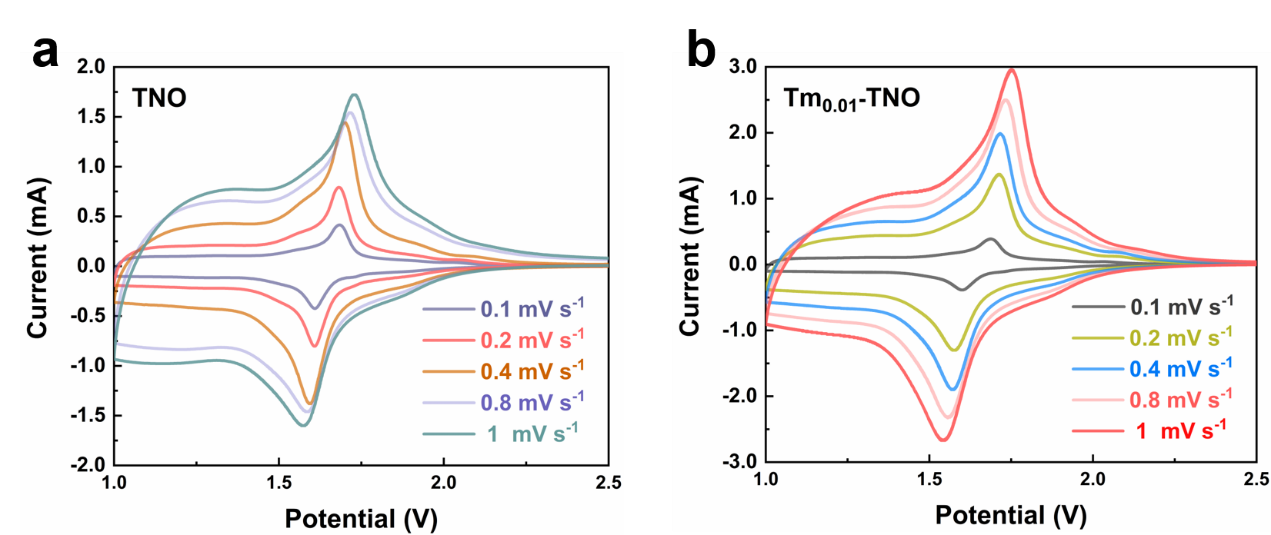


**Figure S5**. CV curves of TNO (a) and Tm_0.01_-TNO (b) electrode at various scan rates.


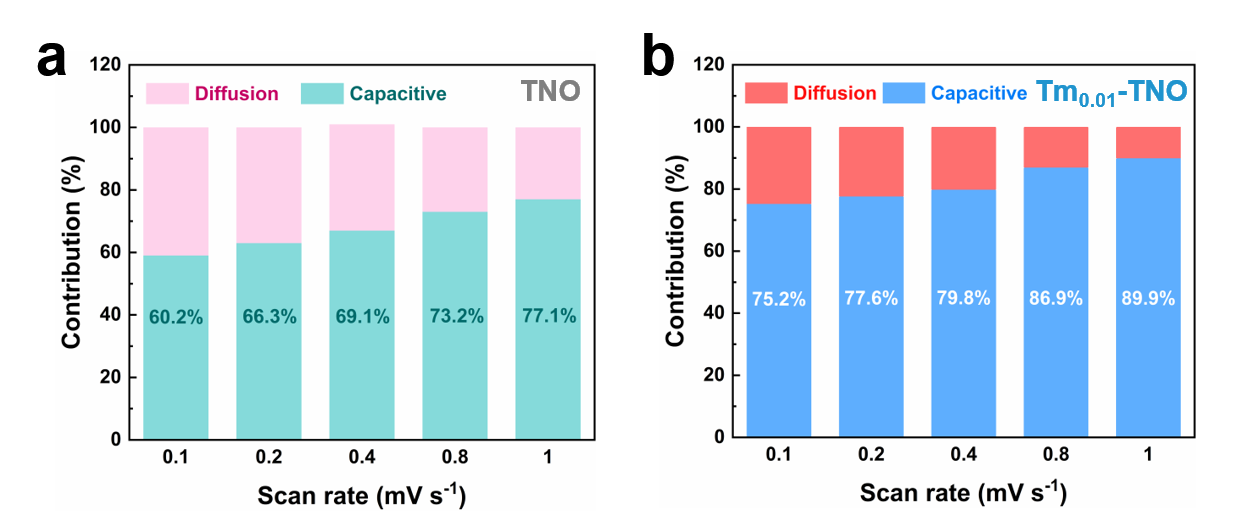


**Figure S6**. The percentage of pseudocapacitive contribution at different scan rates of of TNO (a) and Tm_0.01_-TNO (b).





**Figure S7**. In-situ DRT pattern of TNO.


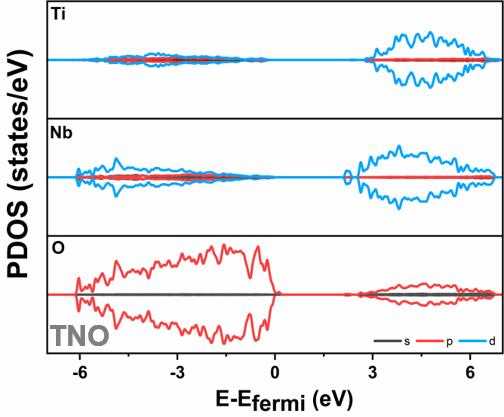


**Figure S8**. Calculated PDOS values of TNO.
